# Ornidazole induced Stevens–Johnson syndrome without body surface involved: A case report

**DOI:** 10.1097/MD.0000000000037164

**Published:** 2023-02-02

**Authors:** Hehe Bai, Xiaodong Wang, Yuanji Wang, Yanhong Li, Weiyan Guo, Juan Lv, Yamei Li, Zhaoqin Hao, Xiaoyan Pan

**Affiliations:** a Department of Pharmacy, Xi’an Central Hospital, Xi’an, PR China; b Department of Ophthalmology, The First Affiliated Hospital of Northwest University, Xi’an, PR China; c Department of Pharmacy, The First Affiliated Hospital of Northwest University, Xi’an, PR China; d School of Pharmacy, Health Science Center, Xi’an Jiaotong University, Xi’an, PR China.

**Keywords:** binoculus reaction keratitis, case report, ornidazole, Steven–Johnson syndrome

## Abstract

**Rationale:**

Ornidazole is a synthetic nitroimidazole derivative that is commonly prescribed for antiparasitic or anti-anaerobic infections. It is generally well tolerated, with known side effects including gastrointestinal tract, anaphylaxis, and central nervous system reactions. Ornidazole-induced binocular reactive keratitis and several mucocutaneous lesions have been rarely reported.

**Patient concerns:**

A 52-year-old woman who suffered from vaginitis and received an ornidazole vaginal plug (0.5 g). Approximately 20 minutes after the suppository was inserted into the vagina, her lips were swollen and valva and labia were burning. Her eyes were red, sore, and watery.

**Diagnosis:**

She was diagnosed as Steven–Johnson syndrome by the ophthalmologist. According to the Naranjo scale, the adverse drug reaction was evaluated to be probable and severe.

**Interventions:**

Dexamethasone was intravenous administrated as anti-inflammatory therapy for 10 days. Eye drops were locally given to relieve edema and promote healing of the epithelium. The symptoms of her eyes, lips, vulva and crissum were soon relieved.

**Outcomes:**

The patient was discharge from hospital with improved symptoms.

**Lessons:**

In order to avoid severe adverse effect, the patient should not use metronidazole ether orally or vaginally. The case emphasized the importance of rapid and accurate diagnosis of Steven–Johnson syndrome induced by ornidazole vaginal plug, especially when the eye symptoms were the chief complaint without body skin involved.

## 1. Introduction

Ornidazole is a synthetic nitroimidazole derivative that is commonly prescribed for antiparasitic or anti-anaerobic infections. It is generally well tolerated, with known side effects including the gastrointestinal tract, anaphylaxis, and the central nervous system reaction. However, there are still some severe adverse reactions, such as hepatitis,^[1,2]^ autoimmune hepatitis,^[3]^ fixed drug eruption,^[4]^ ataxia,^[5]^ and encephalopathy.^[6]^ The suppository is a traditional dosage form, which is usually used for vaginal and anal drug administration, for a much higher local concentration than systematic drug delivery. Ornidazole-induced reactive keratitis and mucocutaneous reactions were rarely reported. Here, the author described a case that used an ornidazole vaginal plug to relieve the symptoms of vaginitis, a series of mucocutaneous reactions that occurred in a short time.

## 2. Case report

A 52-year-old female patient, 160 cm in height and 50 kg in weight, was admitted to the department of ophthalmology with a chief complaint of red, pain, photophobia and watery for 2 days. She had been suffering from vaginitis, and an ornidazole vaginal plug (0.5 g) had been prescribed by the gynecologist. Approximately 20 minutes after she inserted 1 vagunal plug into her vagina, her lips became numb and swollen, the vulva and crissum became itchy, scorching hot and painful. The next day morning, her eyes became red, blurred and lacrimated. The mucous membrane of her mouth was ulcerated and there were several pustules, and the pain worsened. However, her body skin was not involved during the entire process. Before these symptoms appeared, there was no history of ocular or other systemic diseases except for vaginitis. she had no prior history of any medications, diseases or alcohol use, and no record of any drug or food allergies. She was a retired pharmacist and was living at home.

Olopatadine capsules were prescribed by the local dermatologist. However, the symptoms of her eyes, mouth, vulva and crissum were continuing. She still felt that her lips were swollen, and her perineum and anus were burning and painful.

**Figure 1.**
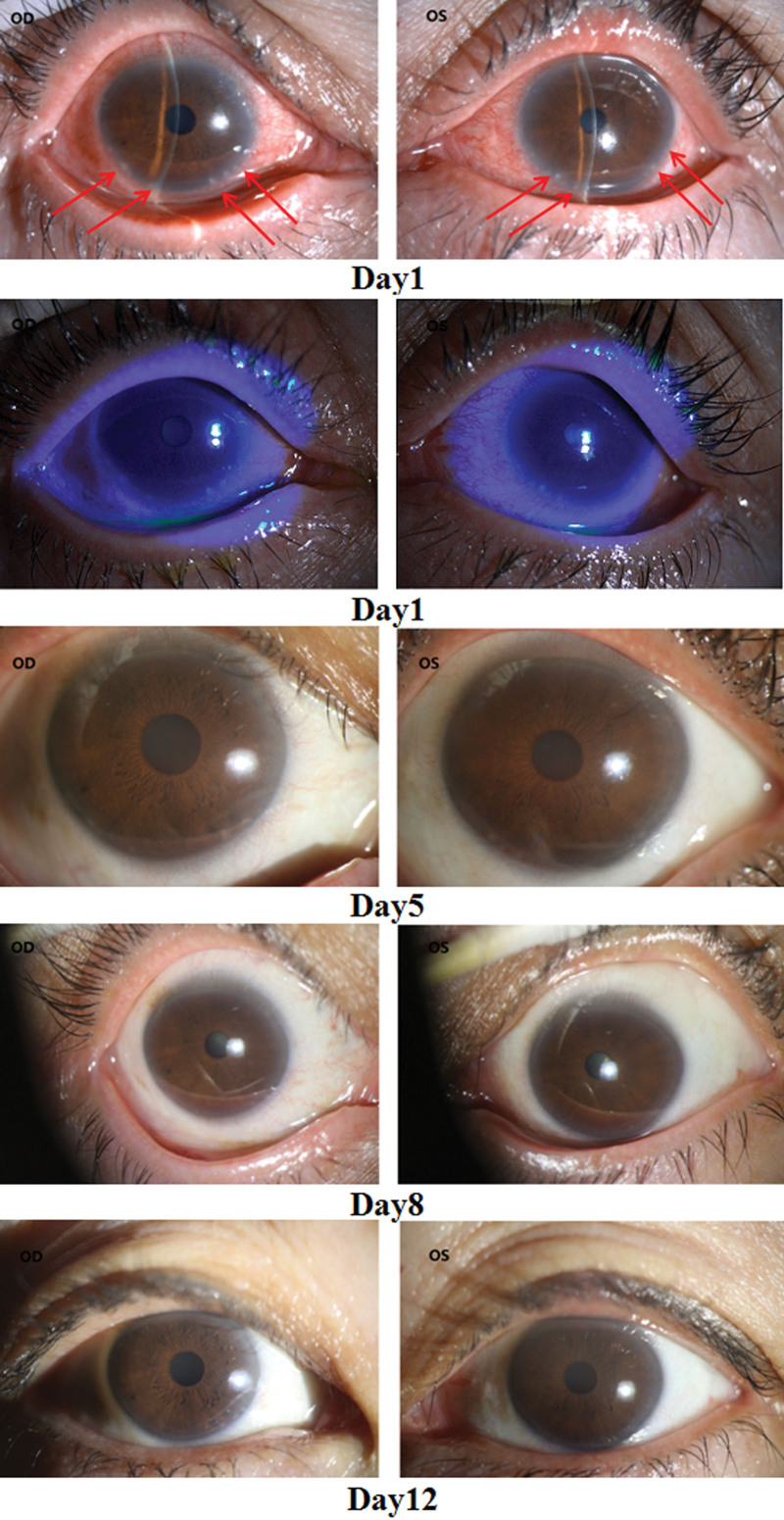
The eye photos of different period were recorded (Instrument Version: Topcon DC4). Both the left and right cornea were edema, several circular gray-white opacity could be seen in the lower limbus (red arrow).

**Figure 2.**
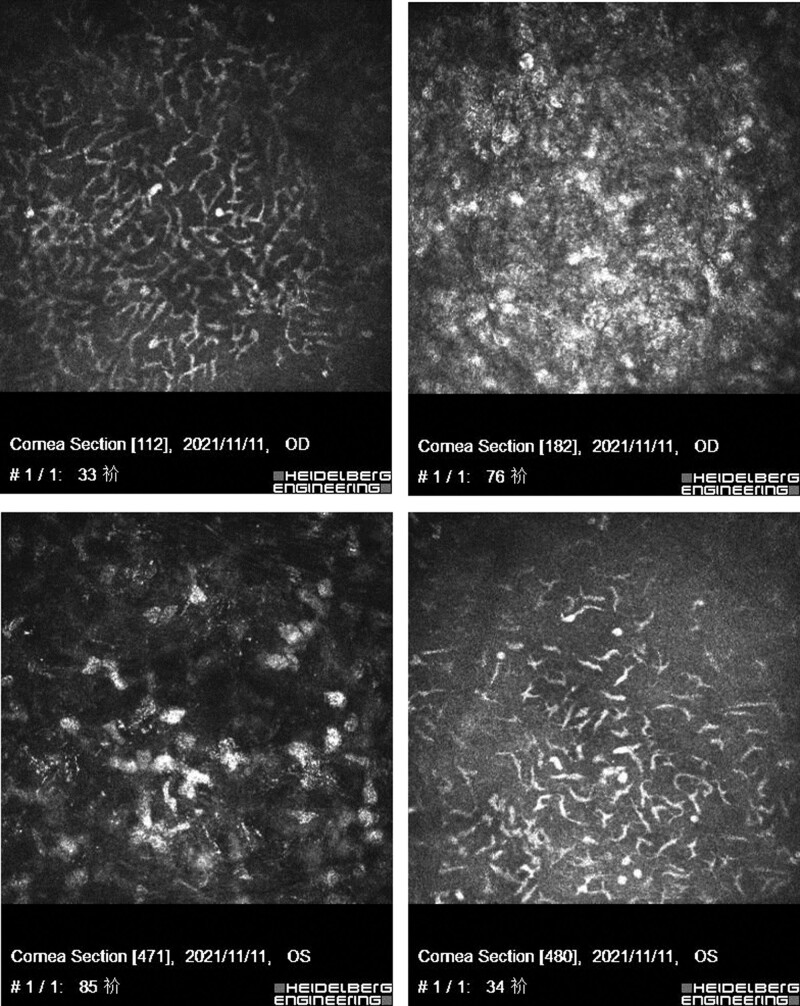
The anterior OCT of both eyes (Instrument Version: Heidelberg Engineering, Spectralis OCT). Punctate opacities change under the front elastic layer was observed. OCT = optical coherence tomography.

The visual acuity was OD 1.0, OS 0.8. The conjunctiva of both eyes was hyperemic. The corneal epithelium was rough, and several round, gray-white opacity areas could be seen on the bottom of the corneal limbus, with a diameter of about 1 mm. Keratic precipitates was positive (++). Photos of different treatment periods were shown in Figure 1. Intraocular pressure was OD 15.0 mm Hg, OS 16.0 mm Hg. Confocal microscopy showed extensive infiltration of inflammatory cells (Fig. 2). Ultrasound indicated mild flocculent opacity in the vitreous bodies of both eyes.

The laboratory examination revealed that the patient had no rheumatism, infectious diseases or any other autoimmune diseases. The functions of her kidney and liver were normal. The patient had no fever during the whole procedure of the disease. Urinalysis and urinary sediment quantization indicated a WBC count of 3+ and occult blood (+−), which prompted the existence of inflammation in the urethra. The hypersensitive C-reactive protein was 3.43 mg/L.

Based on the clinical examination, medical history, and physical examination, our diagnosis was Steven–Johnson syndrome (SJS). It is recommended that the therapeutic schedule include intravenous application of steroids and topical use of antibiotics, steroids, preservative-free artificial tears, and rinsing of the ocular surface, in the acute stages of Steven–Johnson syndrome/toxic epidermal necrolysis.^[7]^ Eye drops, including bromfenac sodium, levofloxacin, prednisolone acetate, tacrolimus, and deproteinized calf blood extract eye gel, were used to improve the symptoms of the cornea and epithelium. Dexamethasone (10 mg) was administrated immediately for 5 days. The symptoms of her eyes, mouth, vulva, and crissum were alleviated. However, she experienced tongue numbness for almost ten days. After 5 days of treatment, her oral mucous membrane completely had recovered. Dexamethasone (5 mg) was used for the next 5 days. Thereafter dexamethasone was discontinued, followed by the administration of 30 mg prednisone acetate tablets. Then the patient was discharged from the hospital, and levofloxacin eye drops were discontinued. She used 20 mg prednisone acetate tablets once a day for the next 7 days. Gradually, 10 mg and 5 mg prednisone were administrated for consecutive 7 days, after which prednisone acetate tablets were discontinued. Olopatadine capsules were totally administrated for ten days. Prednisolone acetate eye drops was gradually decreased and withdrawn 1 month later. Bromfenac sodium eye drops and deproteinized calf blood extract eye gel were used for 1 month. Tacrolimus eye drops were used for almost 3 months.

After 12 days of treatment, the corneas of both eyes became transparent without any edema or crude epithelia. When she left the hospital, her visual acuity was OD 1.2, OS 1.0. The symptoms of her lips, perineum and crissum recovered to normal states. At the same time, vaginitis still afflicted her. The gynecologist prescribed clotrimazole vaginal tablets. After she used this drug, there was no adverse reactions, and the vaginal symptoms disappeared soon.

**Figure 3.**
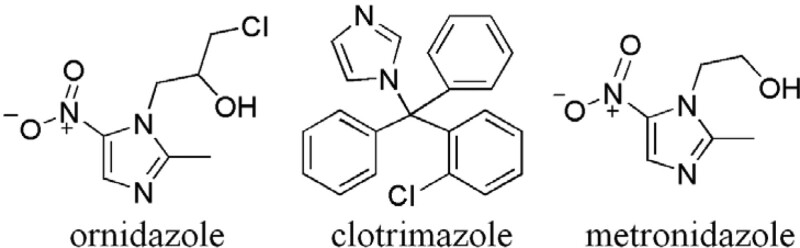
The chemical structures of ornidazole (left), clotrimazole (middle), and metronidazole (right).

The authors hypothesized that adverse effects would occur if the patient used metronidazole, another nitroimidazoles drug which is more usually applied in anti-anaerobic treatment. As the chemical structures of ornidazole, clotrimazole and metronidazole have similar imidazole chemical groups (Fig. 3), they all have an imidazole parent nucleus, while the side chains are comparatively different. These might be the reasons why ornidazole induced binocular reactive keratitis and several mucocutaneous reactions after the administration of the drug by the vagina, whereas clotrimazole did not.

The administration of ornidazole suppository induced binocular reactive keratitis and several mucocutaneous reactions without any body surface involvement, which is quite rare. After systematic analysis, all the injuries were attributed to ornidazole initiated adverse drug reactions. We had evaluated the correlation of administering ornidazole suppository and keratitis using by Naranjo adverse drug reaction evaluation scale, and the total score was 6 (Table 1). The causality between the drug administration and corneal ulceration was probable/likely and severe.

**Table 1:** Naranjo ADR evaluation scale.

Related questions	Score		
	Yes	No	Unknown
5.1. Is there any conclusive report before this ADR?	+1	0	0
2. Do the ADR occur after the use of suspect drugs?	+2√	−1	0
3. Do the ADR relieve after drug withdrawal or use of antagonist?	+1**√**	0	0
4. Does the ADR recur after reuse of the suspect drug?	+2	−1	0
5. Are there other reasons that can cause the ADR independently?	−1	+2√	0
6. Does the ADR repeat after the application of placebo?	−1	+1	0
7. Does the drug reach toxic concentration in blood or other body fluids?	+1	0	0
8. Is the ADR aggravated (relieved) with the increase (decrease) of dose?	+1	0	0
9. Has the patient ever been exposed to the same or similar drugs and had similar reactions	+1	0	0
10. Is there any objective evidence to confirm the reaction?	+1√	0	0
**Total score**	6		

*Note*: The total score ≥ 9 shows that the causal relationship of adverse drug reactions is definite; the total score 5–8 is probably or likely to be relevant; the total score 1–4 is possible to be relevant; the total score ≤ 0 is doubtful to be relevant.

ADR = adverse drug reactions.

Schrodinger 2018-1Maestro (v 115011) QikProp module was used to compare and predict the safety of the 3 compounds. The results were shown in Table 2. Metronidazole and ornidazole could not penetrate the blood–brain-barrier, whereas clotrimazole could. The octanol/water partition coefficients of ornidazole, clotrimazole, and metronidazole indicated that the liposolubility of clotrimazole was the best among the 3 compounds, and the water solubility of metronidazole was the best of them. The order of binding affinity to human serum albumin from high to low was clotrimazole, ornidazole, and metronidazole. The human oral absorption of clotrimazole and ornidazole was better than that of metronidazole. The percentage of human oral absorption of clotrimazole was 100%, while the parameters of ornidazole and metronidazole were 76.6% and 70.1%, respectively.

**Table 2:** The predicted parameters of 3 compounds with Schrodinger software.

Title	CNS	QPlogPo/w	QPlogKhsa	Human oral absorption	Present human oral absorption
Ornidazole	−1	0.656	−0.477	3	76.625
Clotrimazole	1	5.398	1.089	3	100
Metronidzole	−1	−0.019	−0.688	2	70.1

*Note*: CNS predicted central nervous system activity on a −2 (inactive) to +2 (active) scales.

QPlogPo/w predicted octanol/water partition coefficient. The larger the fat-water partition coefficient, the more easily soluble in fat and vice versa, conversely the more easily soluble in water.

QPlogKhsa predicted the binding ability to human serum albumin.

Human oral absorption predicted the qualitative human oral absorption: 1, 2, or 3 for low, medium, or high.

Present human oral absorption predicted human oral absorption on 0 to 100% scale.

Based on the results of QikProp properties and the patient’s drug administration history, the pharmacist suggested that the patient should not use ornidazole ether via intravenous, oral or vaginal administration. As the chemical and biological characteristics of metronidazole and ornidazole were so similar, the patient should not use metronidazole ether to avoid severe adverse effects. The limitation of the present case was that there were no relevant results of genetic testing.

## 3. Discussion

The present case suggests that ophthalmologists play a key role in the diagnosis and treatment of patients whose main symptoms were induced by drugs, especially when the eyes were involved, with or without the mucous membrane of the body skin involved.^[8]^ SJS is an immune-mediated disease that is usually induced by drug intake.^[9]^ The eyes are affected in 40% to 80% of SJS cases.^[7]^ Acute ocular involvement is reported to occur in 50% to 88% of patients with SJS. Antibiotics are the most common cause of death in almost half of patients.^[7]^ SJS always involves cutaneous and mucosal symptoms, with nonspecific symptoms such as fever, rhinitis, headache, conjunctivitis and sore throat. The most common complications are infections, involving the eye, mouth pharynx, esophagus, and genitourinary tract.^[10]^ Sometimes atypical SJS might not manifest on the body skin, as in the present case, which may mislead doctors to consider other diagnoses rather than SJS, especially when more than 1 tissue or organ is involved. Topical antibiotics are recommended to prevent such infections.^[8]^

Özkaya^[11]^ reported a case of a 78-year-old Turkish woman who experienced purpuric plaques after using ornidazole for a recurrent genitourinary infection. The trunk, arms and legs were all involved. Histopathological examination revealed interfacial dermatitis and leukocytoclastic vasculitis. These results indicate that ornidazole could induce immune-related reactions in different forms.

Other drugs can also induce immune-related adverse effects, such as PD-1/PD-L1,^[12,13]^ chemotherapeutics, and antibiotics. Some immune-related reactions manifest on the ocular surface, such as uncontrolled inflammation, dry eye disease, epithelial keratitis, stromal ulceration and corneal perforation.^[14]^ Thomas et al^[15]^ reported a case of an 88-year-old Caucasian man with metastatic melanoma, who was administered pembrolizumab at 2 mg/kg. After 24 months of pembrolizumab therapy, he developed corneal erosions, oral and genital ulcerations, a syndrome similar to Behcet disease. These symptoms of this Caucasian patient were resemblance to those we have reported in this study.

However, as the chemical structure of different nitro-imidazole compounds had the same imidazole group, their adverse effects might be common, especially when the reaction was manifested on the cornea. In the present case, the patient experienced cornea and several skin mucous membrane adverse effects after she used an ornidazole vaginal plug. Vaginitis that afflicted her may have recurred in the future. However, ornidazole was no longer recommended. She used clotrimazole vaginal plug twice and no adverse effects occurred. It was difficult to predict whether the similar reactions would occur when metronidazole was used. Based on this consideration, the Schrodinger 2018-1Maestro (v 115011) QikProp module was used to predict the safety of ornidazole, clotrimazole, and metronidazole. These results indicated that ornidazole should not be used either orally or via vaginal drug administration. Additionally, metronidazole should be closely monitored when she uses it in the future.

## 4. Conclusions

Ornidazole is a commonly used nitroimidazole derivative that may cause multiple skin and mucous membrane reactions, and the symptoms are always similar to anaphylaxis, especially when it is used for the first time. The route of administration may be closely related to adverse effects such as vaginal drug delivery. The local concentration is much higher in the local mucous membrane than in the blood. Doctors should pay more attention to drug induced adverse effects, especially when the symptoms involve more than 1 system, with or without the body surface being involved. Early recognition and appropriate treatment are quite important, and promptly drug withdrawal may prevent further damage. This case emphasize the importance of rapid and accurate diagnosis of multiple skin and mucous membrane reactions induced by the ornidazole vaginal plug, especially when the body skin is not involved. However, for this patient, long term follow-up is necessary. The patient was extremely satisfied with the treatment outcome, as the symptoms of both her eyes and vaginitis had completely disappeared. She was content to have been given enough attention throughout the treatment.

## Author contributions

**Conceptualization:** Hehe Bai, Yuanji Wang, Weiyan Guo, Xiaoyan Pan.

**Data curation:** Xiaodong Wang, Zhaoqin Hao.

**Formal analysis:** Weiyan Guo, Juan Lv.

**Funding acquisition:** Hehe Bai, Xiaodong Wang.

**Investigation:** Xiaodong Wang, Yuanji Wang, Yanhong Li, Yamei Li.

**Methodology:** Hehe Bai, Yanhong Li, Weiyan Guo, Yamei Li, Xiaoyan Pan.

**Project administration:** Juan Lv.

**Resources:** Yuanji Wang, Weiyan Guo, Juan Lv.

**Software:** Yanhong Li, Xiaoyan Pan.

**Supervision:** Yanhong Li, Yamei Li, Zhaoqin Hao, Xiaoyan Pan.

**Validation:** Hehe Bai, Xiaodong Wang, Yuanji Wang, Weiyan Guo.

**Visualization:** Zhaoqin Hao.

**Writing – original draft:** Hehe Bai.

**Writing – review & editing:** Yuanji Wang.
